# Using eclipse scripting to fully automate in‐vivo image analysis to improve treatment quality and safety

**DOI:** 10.1002/acm2.13585

**Published:** 2022-03-22

**Authors:** Ananta Raj Chalise, Casey Bojechko

**Affiliations:** ^1^ Department of Radiation Medicine and Applied Sciences University of California San Diego San Diego California USA

**Keywords:** automated comprehensive QA, EPID, ESAPI, Halcyon, patient specific QA, Quality assurance

## Abstract

**Purpose:**

An automated, in‐vivo system to detect patient anatomy changes and machine output was developed using novel analysis of in‐vivo electronic portal imaging device (EPID) images for every fraction of treatment on a Varian Halcyon. In‐vivo approach identifies errors that go undetected by routine quality assurance (QA) to compliment daily machine performance check (MPC), with minimal physicist workload.

**Methods:**

Images for all fractions treated on a Halcyon were automatically downloaded and analyzed at the end of treatment day. For image analysis, compared to first fraction, the mean difference of high‐dose region of interest is calculated. This metric has shown to predict changes in planning treatment volume (PTV) mean dose. Flags are raised for: (Type‐A) treatment fraction whose mean difference exceeds 10%, to protect against large errors, and (Type‐B) patients with three consecutive fractions with mean exceeding ±3%, to protect against systematic trends. If a threshold is exceeded, a physicist is e‐mailed, a report for flagged patients, for investigation. To track machine output changes, for all patients treated on a day, the average and standard deviations are uploaded to a QA portal, along with the reviewed MPC, ensuring comprehensive QA for the Halcyon. To guide clinical implementation, a retrospective study from November 2017 till December 2020 was conducted, which grouped errors by treatment site. This framework has been used prospectively since January 2021.

**Results:**

From retrospective data of 1633 patients (35 759 fractions), no Type‐A errors were found and only 45 patients (2.76%) had Type‐B errors. These Type‐B deviations were due to head‐and‐neck weight loss. For 6 months of prospective use (345 patients), 13 patients (3.7%) had Type‐B errors and no Type‐A errors.

**Conclusions:**

This automated system protects against errors that can occur in vivo to provide a more comprehensive QA. This fully automated framework can be implemented in other centers with a Halcyon, requiring a desktop computer and analysis scripts.

## 1 INTRODUCTION

Electronic portal imaging devices (EPIDs), primarily used for pretreatment checks in intensity‐modulated radiation therapy (IMRT) and volumetric‐modulated arc therapy (VMAT), are gaining use for in‐vivo dosimetry.^1^, ^2^ Studies analyzing the effectiveness of EPIDs in detecting clinically reported incidents have found that when used for pretreatment quality assurance (QA), EPIDs detect a low number of clinically reported incidents (6%).^3^ However, they are much more effective when used in‐vivo QA. Same study reported that EPIDs are able to detect 74% of incidents when used for the first fraction and 91% when all fractions are considered.

While the usage of EPIDs for in‐vivo dosimetry has been increasing, it still has not become mainstream due to an increase in workload and ambiguity on how to interpret and utilize in‐vivo data.^4^ Recently, publications have reported the clinical use of a commercially available automated system for EPID‐based dosimetry^5^, ^7^ (PerFRACTION™, part of SunCHECK™, Sun Nuclear Corporation, Melbourne, FL, USA). Olch et al. used the first treatment fraction as a baseline to compare other fractions and were able to identify changes in anatomy, patient setup as well as external device position for their study of 855 fractions.^7^ Bossuyt et al. have reported using absolute dose prediction, where they predict EPID images and compare them to delivered EPID images instead of comparing to baseline, for more than 8600 fractions.^5^ All these studies used gamma comparison‐based metrics to determine pass/fail for evaluating their treatment fractions.

While gamma pass rate is effective in detecting a difference between two dose distributions, it is not effective in discerning clinically actionable information.^8^, ^9^, ^10^, ^11^ This requires tolerance levels to be empirically optimized based on clinical experience which can vary between treatment site and clinic. Using a different metric, gradient‐dose segmented analysis (GDSA),^12^, ^13^ our group has analyzed in‐vivo transit EPID images where mean image differences are calculated from the pixels in the high‐dose, low gradient region using the first fraction as the baseline. This high‐dose, low gradient region roughly corresponds to the projection of planning treatment volume (PTV) onto the imaging panel.^13^ This work illustrated the use of the GDSA method to provide a simple metric to compare transit EPID images to predict changes in mean PTV dose (PTV *D*_mean_) for detecting treatment errors, as well as the patient changes over the course of treatment. Additionally, detecting changes in machine output can be achieved by averaging these metrics for all patients treated on a given day and tracking over time.^14^

The purpose of this work is to present an automated EPID QA framework for the Varian Halcyon. This approach neither requires a complex commissioning process nor intensive computing resources, making it easily portable to other clinics with a Halcyon. Furthermore, this tool can be ported to other Varian Linac types, the only additional requirement is that the EPID arm is extended and the data needs to be collected during the treatment. Monitoring every fraction of treatment gives quantitative data for the variation in treatment delivery.

## 2 METHODS

The Varian Halcyon linear accelerator is unique as it collects in‐vivo images by default through its Varian aS1200 digital megavoltage imaging panel (Varian Medical Systems, Palo Alto, CA). This imaging panel is mounted directly opposite to single energy 6 MV‐flattening filter‐free source.^15^ The imager has a fixed 154 cm source‐to‐imager distance, 43 × 43 cm physical size, 28 × 28 cm isocentric projection, and 1280 × 1280 pixels image matrix. Given its size and position, complete image data are collected for all treatment fields. Image data are automatically exported to the record‐and‐verify system ARIA (Varian Medical Systems). The images are calibrated to calibrated units (CUs) which are defined as 1 CU per 1 Monitor unit (MU) for a standard field size of 10 × 10 cm with no patient or phantom present. CUs are a relative calibration factor that encapsulates the response from the detector and are not an absolute measurement of the dose. Darkfield, as well as flood field corrections, are applied before these calibrations. The output measured by the EPID is recorded daily with the machine performance check (MPC) (Varian Medical Systems).

**FIGURE 1:**
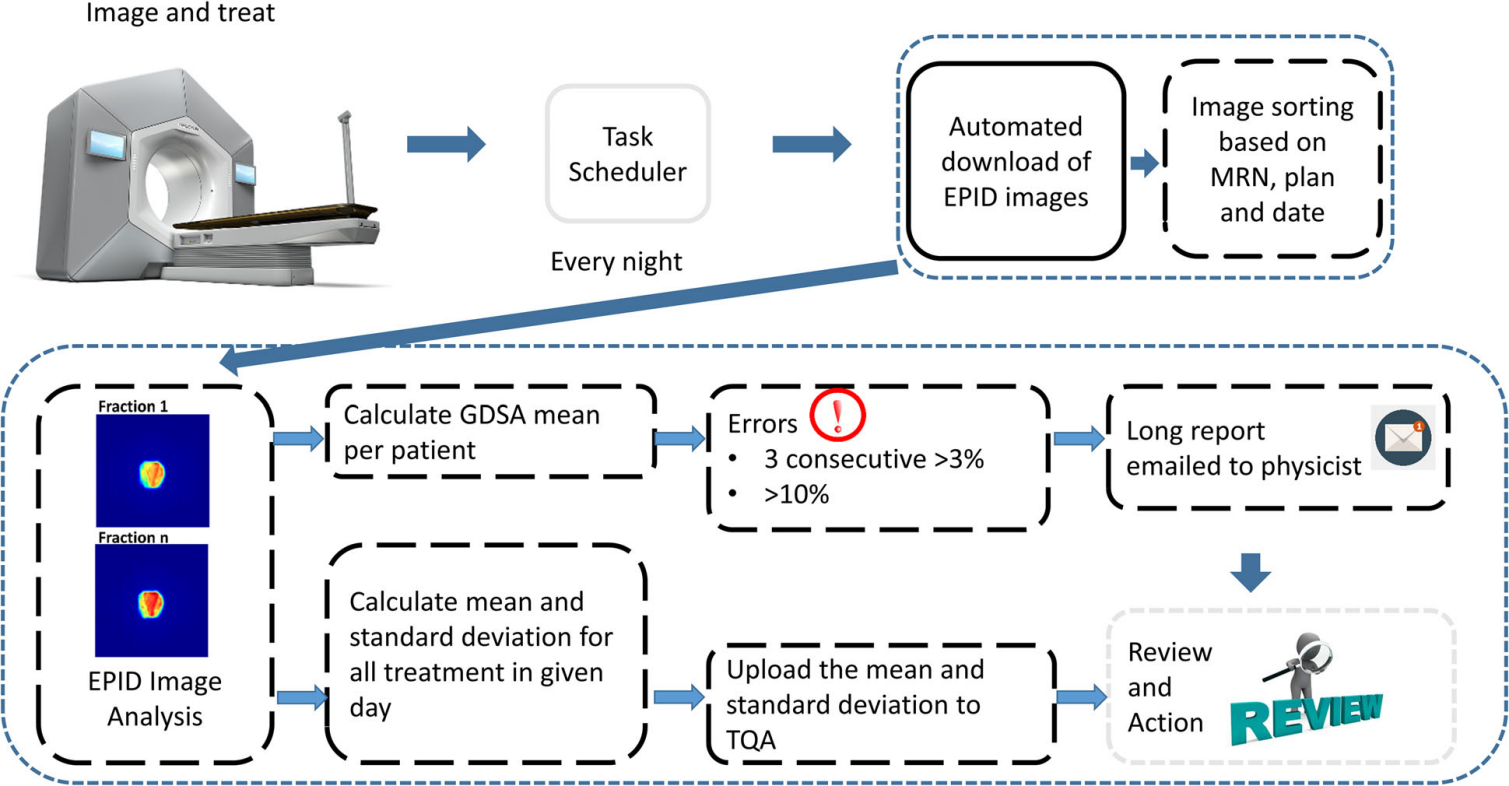
Workflow of codes to automate quality assurance (QA) with postscript (grey solid box), C# (black solid box), and MATLAB (black broken box) with minimal human intervention (grey broken box).

A combination of scripts in post‐script, C#, and MATLAB was used to download EPID images. These images were processed automatically at the end of the treatment day. The workflow is illustrated in Figure 1. Every night, a task scheduler written in post‐script performs two main tasks: download images and image analysis.

The list of patients treated on the Halcyon on a given day is autogenerated using ARIA reports. A C# executable reads the patient list and automatically downloads the day's in‐vivo EPID images. In this executable, the code was configured to download only RTIMAGE type of image modality. MATLAB code then sorts all these EPID images based on the medical record number, plan, and date. This C# executable is adapted from an open‐source EvilDICOM (https://github.com/rexcardan/Evil‐DICOM), which gives easy access to Digital Imaging and Communication in Medicine (DICOM) files in ARIA. The computer running these scripts must be connected to the database server and whitelisted by the administrator. The details of setting up the DICOM daemon, network nodes used for receiving and sending the DICOM messages, are illustrated in the Varian API handbook.^16^ The essential information from the daemon, which needs to be incorporated in the executables is the application entity title, Internet protocol address, and port through which it will communicate to the server. This executable makes the usage of the Eclipse Scripting API (ESAPI), together with DCMTK, an open‐source package to batch process‐desired objects as DICOM files to a configured location.

After the download and sorting of DICOM images, additional MATLAB code then performs the GDSA analysis, where the mean is computed for each fraction for each patient. For each treatment day the GDSA mean is computed for each fraction/patient treated. The average and standard deviation are calculated from the resultant distribution; the average gives an estimate in the change in machine output and the standard deviation gives an estimate of the patient variability on a given day.

Flags are raised for patient‐related errors and machine‐related errors.

Patient‐related errors:

- Patient has one fraction with a deviation of ± 10% to prevent large errors (Type‐A).
- Patient has three consecutive fractions with a deviation of ± 3% to prevent systematic trends (Type‐B).

Machine‐related errors:

- If the mean between two consecutive treatment days differs by 1%.

Every time a flag is raised, the system also generates a report consisting of images and statistics of the patient and is e‐mailed to a physicist for review.

The calculated values are also automatically uploaded to a web‐based QA portal, Total QA (Image Owl, Greenwich, NY) after clinical hours to give a summary of the day. These results are checked by the physicist of the day the following morning and are alerted to any exceeded tolerances.

To aid in the implementation of this system a retrospective study was performed to assess typical clinical variation as well as any variations over time. Patients treated were assessed retrospectively on a monthly basis. For each month of the treatment, deviations were identified and categorized by treatment site.

After developing and validating this automatic comprehensive QA tool, its usage was tested for head‐and‐neck cases to see if the in‐vivo data collected can aid in decision making when a patient needs replanning due to anatomy changes. Currently, in our clinic, the decision to replan a head‐and‐neck case is based on the physician's review of daily imaging. We compared this approach to using a GDSA mean exceeding a threshold to trigger a replan. The number of replans (52 out of 182 head‐and‐neck patients) performed in the clinic was compared to a flag found by the GDSA mean. Using the physician's replanning as ground truth we assessed the sensitivity and specificity of our method. Additionally, since the triggers for deciding to replan can be qualitative, we explored the correlation between the GDSA mean and anatomical changes. There are several metrics to quantify the change in the outer body contour for head‐and‐neck treatments.^17^ We used the maximum reduction of the outer body contour at the isocenter level to quantify the change in the outer contour.

## 3 RESULTS

### 3.1 Retrospective analysis

**FIGURE 2:**
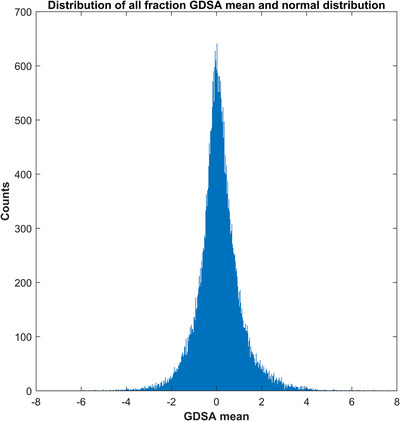
Gradient‐dose segmented analysis (GDSA) means distribution for all fractions (*N* = 35 759) treated from November 2017 to December 2020.

In order to guide our prospective utilization, a retrospective analysis for the Institutional Review Board‐approved study period from November 2017 till December 2020 was conducted. During this period, a total of 1633 patients were treated with 35 759 fractions. The overall GDSA mean and standard deviation for this study period were found to be 0.12% and 1.00%, respectively. The distribution for the GDSA means for all fractions is shown in Figure 2.

The data were analyzed by the treatment site, divided into abdomen/pelvis, head‐and‐neck, brain, chest/lung, and extremities. Table 1 shows the GDSA mean and standard deviation per treatment site. The number of fractions that exceeded a threshold of 3% is also shown.

**TABLE 1:** Site‐specific distribution of retrospective data

Site	Patients	Fractions (% total)	Mean GDSA_μ_(%)	Standard deviation of GDSA_μ_.	*n* Fractions │GDSA_μ_│ > 3%
Abdomen/pelvis	671	17 134 (48)	–0.02	0.86	126
Head‐and‐neck	390	8909 (25)	0.34	1.15	237
Brain	283	5393 (15)	0.07	0.57	8
Chest/lungs	254	3833 (11)	0.34	1.46	206
Extremities	35	490 (1)	–0.26	1.19	20

Table 1 gives the mean, standard deviation, and the number of fractions exceeding 3% for five major sites: abdomen/pelvis, brain, chest/lungs, extremities, and head‐and‐neck. As it is evident from the table, chest/lungs and head‐and‐neck treatments have more fractions exceeding the ±3% threshold because of the tumor shrinkage/weight loss in these sites.

**FIGURE 3:**
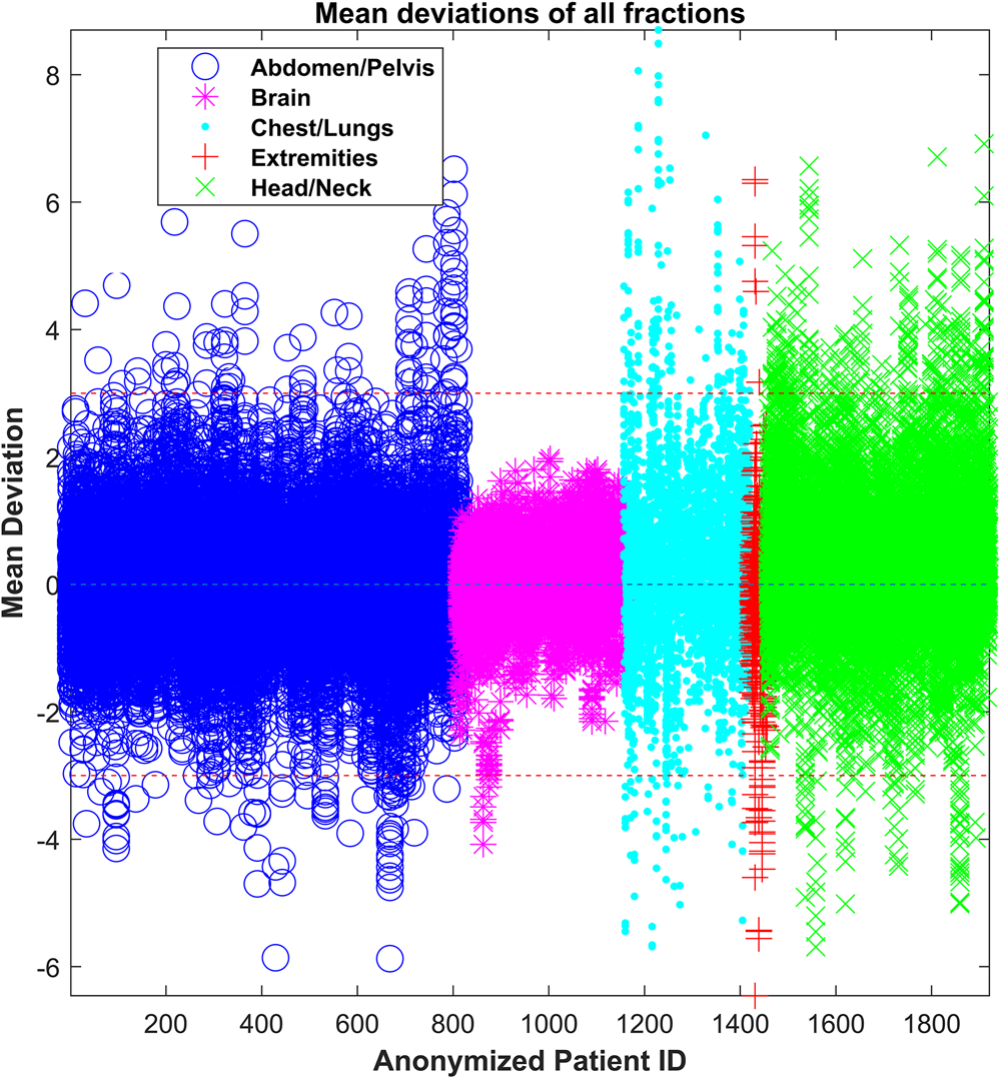
Mean value for all fractions of all anonymized patients, grouped by treatment site where each patient is shown as a point in a vertical line and each point in a vertical line represents various fractions for that patient.

Figure 3 shows all patients color‐coded by treatments site along with ±3% threshold lines. Each point in a vertical line represents various fractions for that patient. It is seen that the majority of these fractions (98.33%) are within the ±3% threshold. The threshold is most commonly exceeded for chest/lungs and head‐and‐neck treatment as outlined in Figure 3 and Table 1. The cases with abdomen are random deviations seen due to changes in bowl filling. For the brain case, it was a treatment for the right orbit where the PTV was right next to sinus cavity which had different filling over the course of treatment. Out of 1633 patients, 45 patients (2.7%) had type‐B errors.

**FIGURE 4:**
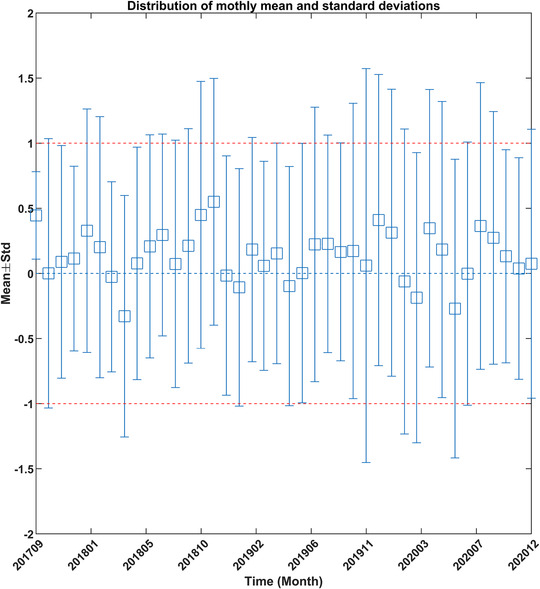
Mean gradient‐dose segmented analysis (GDSA) value for all fractions delivered per month. Error bars are the standard deviation for the monthly distribution.

**FIGURE 5:**
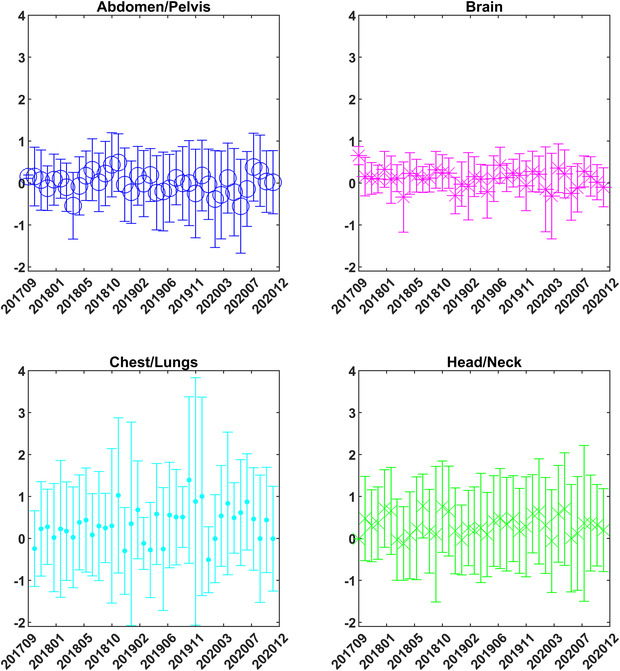
Gradient‐dose segmented analysis (GDSA) mean distributions of four major sites over various months (smaller *y* range for abdomen/pelvis and brain).

**FIGURE 6:**
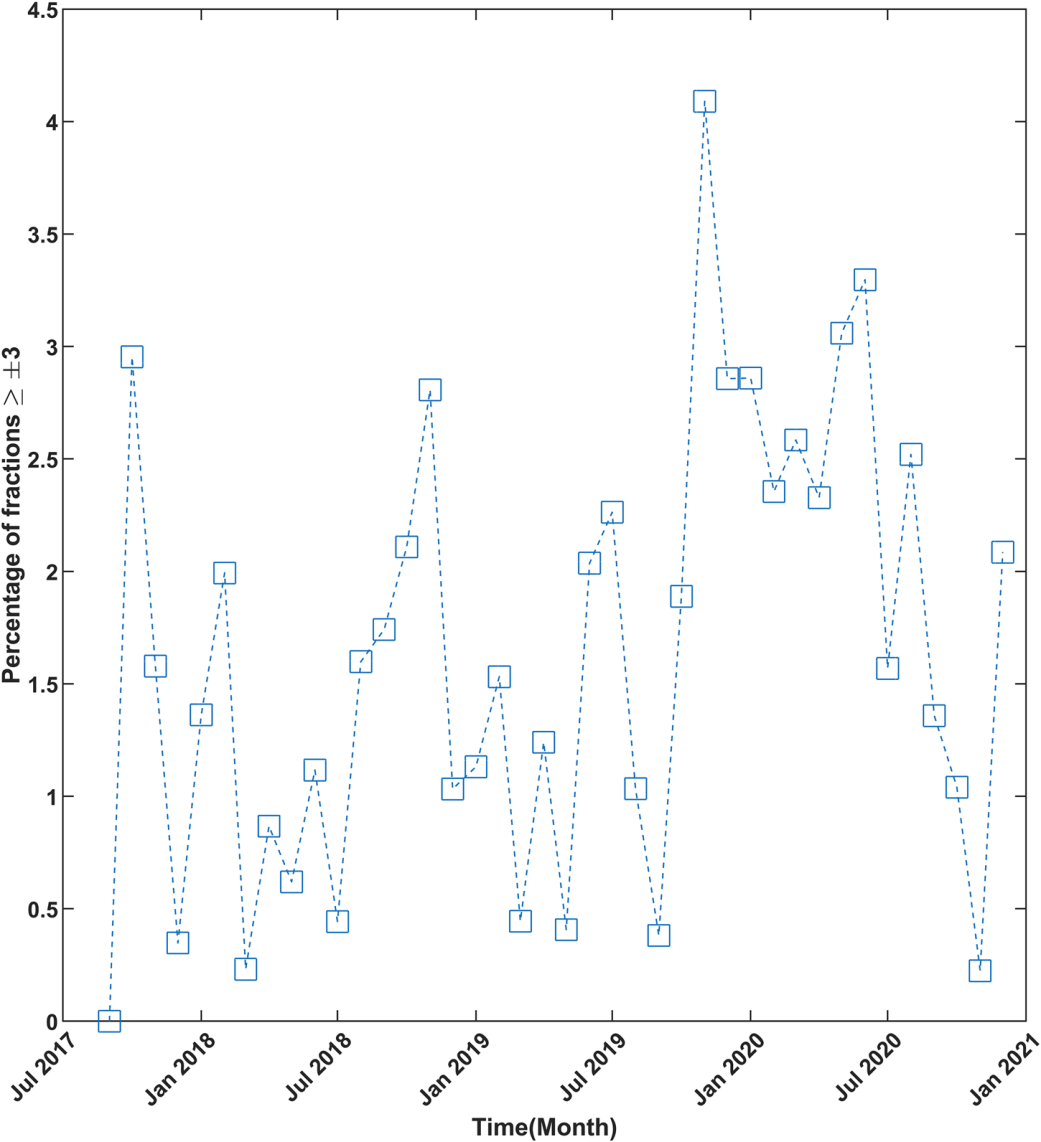
Monthly distribution of the percentage of fractions treated in the month exceeding the ±3% gradient‐dose segmented analysis (GDSA) mean.

**FIGURE 7:**
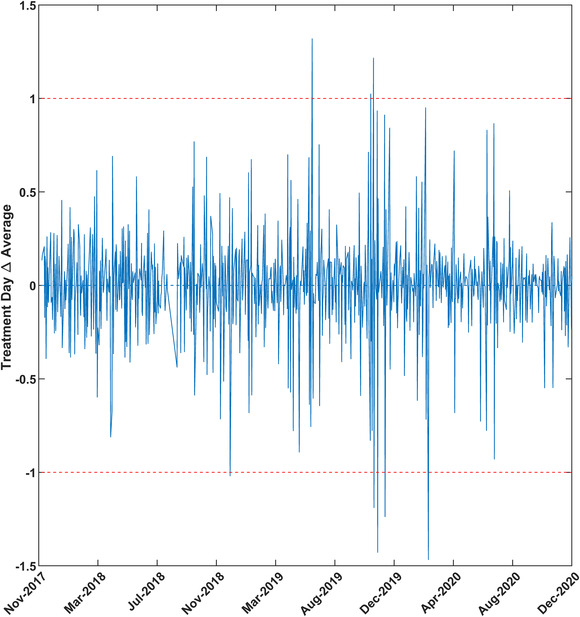
Daily averages of all treatment fractions for the given day.

To check the stability of treatment over time, the mean and standard deviation was analyzed for all fractions treated in a given month. The monthly mean and standard deviations are shown in Figure 4. In this figure, the months with higher deviations indicate that more number of fractions exceeded the threshold. Figure 5 details the monthly mean of four main treatment sites. The smallest deviation can be seen in the case of the brain followed by the abdomen. As mentioned earlier, chest/lungs and head‐and‐neck treatments have more deviations originating from the fact that these sites have greater anatomical variability from tumor shrinkage and weight loss. These changes were assessed qualitatively only and quantitative analysis will be the subject of future study. Figure 6 shows the percentage of fractions treated exceeding 3% per month. The month with the highest number of fractions exceeding the threshold was November 2020, followed by June 2020. The cause of these higher rates of deviations was driven by a small number of patients (one or three patients) with anatomical variations in a given month. These patients were either chest/lung or head‐and‐neck treatments. Figure 7 shows the difference of consecutive daily mean averaged over all patients treated on a given day. Deviations of greater than 1% were investigated. In total, seven instances of the daily average exceeding ±1% were found. Three of these were due to a TG‐51 output adjustment or imager recalibration. The other four instances were due to a small number of patients skewing the daily distribution.

### 3.2 Prospective usage

Prospective usage began in January 2021. In 6 months of study, the system raised a Type‐B flag for 13 patients (3.7%) out of 345 patients. This is comparable to the rate of Type‐B cases for the retrospective data set, 2.76%. Out of 13 patients flagged, head‐and‐neck were dominant (seven cases) followed by abdomen/pelvis and chest/lungs (three cases and two cases, respectively), and extremities were flagged only once. All the head‐and‐neck patients showed loss of weight and/or tumor shrinkage. No Type‐A cases (deviations > 10%) were identified. Two instances of difference in daily average mean exceeding 1% were observed, one of which was due to a TG‐51 recalibration and the other was due to a small number of patients skewing the daily distribution.

#### 3.2.1 Head‐and‐neck case study

For both retrospective as well as prospective usage, head‐and‐neck patients were found to have the most anatomical deviations when compared to other sites. These larger deviations initiated a deeper study of this site to determine if the in‐vivo measurements could be used as a trigger for a replan. Sensitivity and specificity analysis was performed with five variations of the GDSA threshold. The frequency of fractions exceeding thresholds were evaluated. Thusly evaluated patients were categorized into true positive, false positive, false negative and true negative based on physician's decision to replan as a “ground truth” to calculate sensitivity and specificity as outlined in Table 2.

**TABLE 2:** Sensitivity and specificity for different cases

Case	True‐positive	False‐negative	False‐positive	True‐negative	Sensitivity	Specificity
A	10	38	28	105	0.2	0.78
B	33	19	64	66	0.63	0.50
C	28	24	50	80	0.53	0.61
D	28	24	37	93	0.53	0.71
E	33	19	51	79	0.63	0.60

*Note*: A: One fraction > 3%.

B: One fraction > 2%.

C: Two fractions > 2%.

D: Two fractions > 2% but not last 5 fractions.

E: One greater fraction than 2% but not last 5 fractions.

**FIGURE 8:**
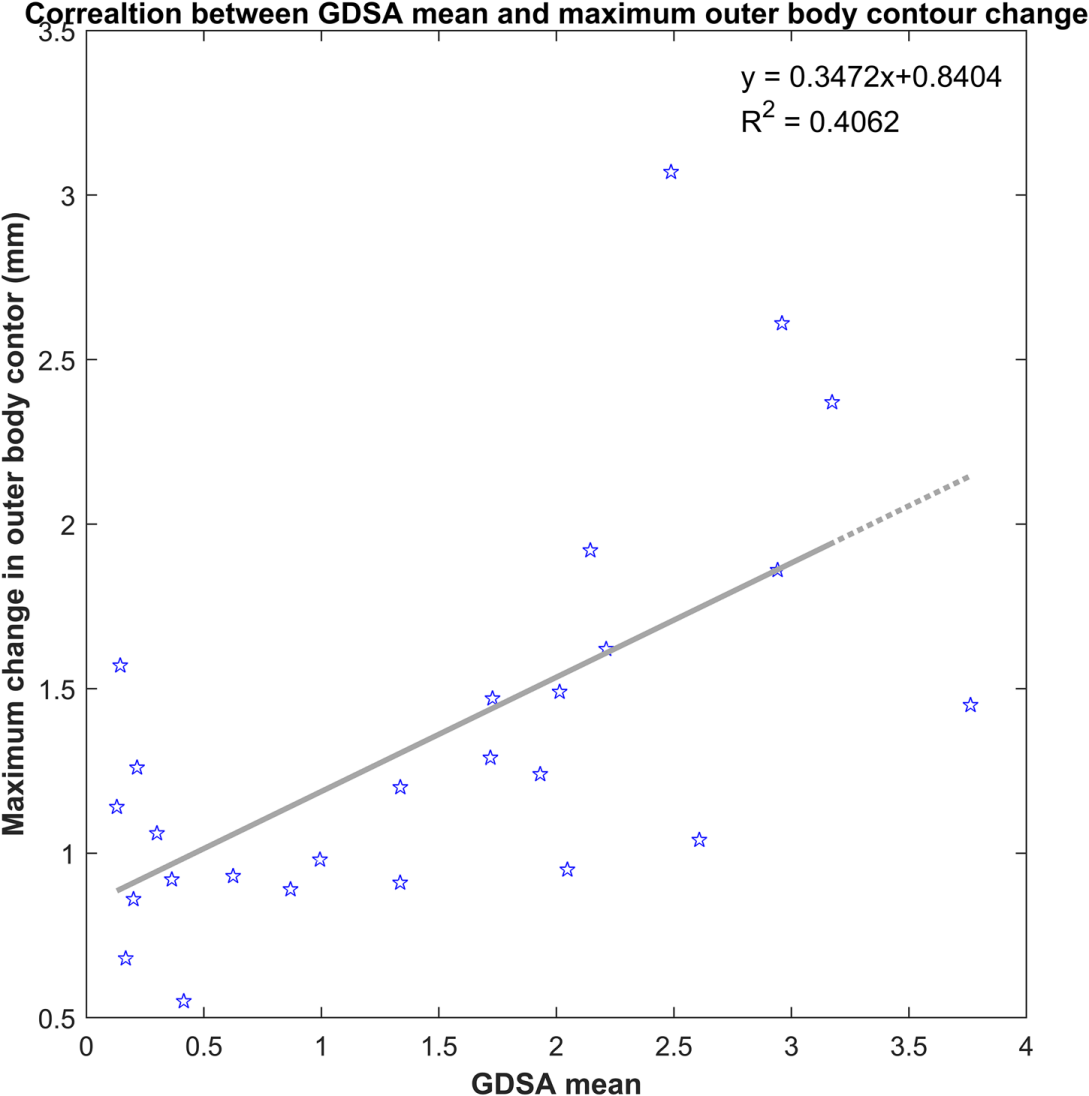
Correlation between gradient‐dose segmented analysis (GDSA) mean and maximum body shrinkage at isocenter level.

It was found that using the GDSA mean > 2%, excluding the last five fractions, we were able to identify physician‐initiated replanning cases with a sensitivity of 0.63 and specificity of 0.60. We also analyzed the correlation between the GDSA changes in outer body contour. The relationship between the GDSA mean and the maximum change in the outer body contour was weakly correlated, *R* ^2^ = 0.41, and is shown in Figure 8.

## 4 DISCUSSION

The QA tool that we have developed is a fully automated system that is using in‐vivo data from every fraction of treatment. This system can track the variability of patient treatments and will alert a physicist when a predetermined threshold is exceeded. No other intervention is required from the medical physicist, limiting the additional workload needed to perform the QA. The analysis of a large number of patients retrospectively (1633) has informed our prospective use of this tool.

The sites which crossed ±3% threshold were prominently head/neck and chest/lungs due to tumor shrinkage rather than small setup errors in these regions. Previous analysis of in‐vivo EPID systems has shown that patient shifts are not readily detected, especially in the treatment of soft tissue.^18^

Using the in‐vivo data from every fraction of treatment has verified that our clinical operations are stable, observing that the vast majority of fractions treated had small deviations (Figure 1) and from month‐to‐month treatments remained stable. Also, it is comforting to verify that no large error (> 10%) was recorded over years of treatment.

**FIGURE 9:**
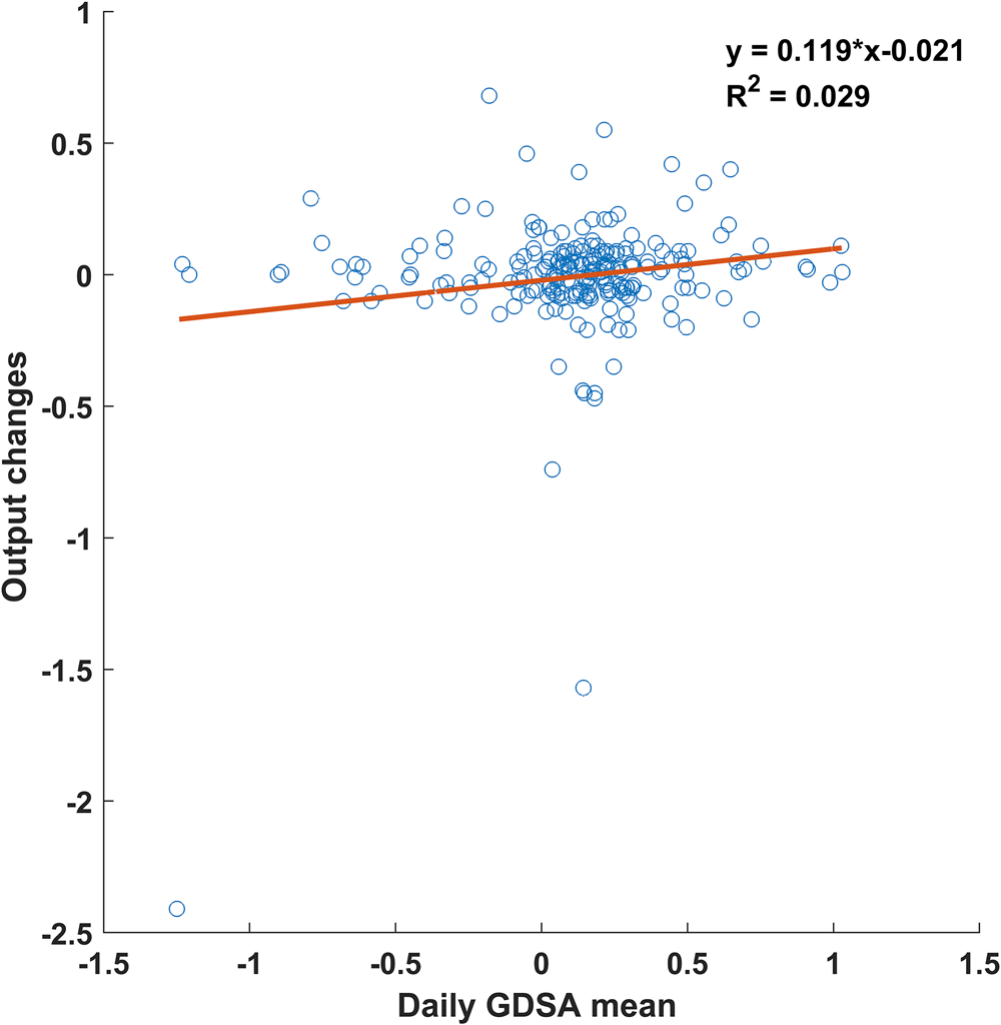
Correlation between daily gradient‐dose segmented analysis (GDSA) mean and output changes between consecutive days.

By measuring the change in the daily mean, we were able to detect changes in output caused by TG‐51 recalibration and imager recalibrations. It also gave a number of false positives due to patient deviations. However, the rate of 1 recorded deviation per 4 months does not add a significant workload and protects against large sudden changes in output. There was no correlation (*R* ^2^ = 0.029) observed between the change in output measured by the MPC between two consecutive days and in‐vivo EPID system for small changed in dose; however, for large deviations in MPC recorded dose (> 2.4%) large deviations were also seen for in‐vivo GDSA measurements as shown in Figure 9. The other larger deviation in output changes (−1.57%) in Figure 9 was not picked up by GDSA due to imager recalibration.

Threshold of ±10% for Type‐A errors was set to only be triggered by a large error such as a treatment machine malfunction. This threshold was set larger than the maximal deviation observed from patient anatomical variations. The threshold for Type‐B errors (three consecutive fractions > ±3%) was guided by the retrospective study (Figure 3) where majority of the fractions were within ±3%. If three consecutive fractions exceeding ±2% were considered, the system would have raised 31 flags (8.98%) and 144 flags (8.81%) for the prospective and retrospective study, respectively. This value is not absolute and can be varied based on clinical goals. For the threshold for daily averages, a value of a 1% deviation between two consecutive days was set based on earlier work that found our system was able to detect output deviations on the order of 1%.^14^ Additionally it is our clinical goal to control output on the order of 1%.

In addition to verifying stability, this tool can be used to detect any clinical deviations and further improve clinical operations by identifying sources that contribute to the variance of treatment. For instance, the retrospective study using this tool has shown that the treatment site with the largest variance is head‐and‐neck followed by chest/lung, and hence more efforts can be made to reduce anatomical variations for these sites.

When using this tool as a flag for head‐and‐neck treatments it has been shown to have marginal sensitivity and specificity when compared to the physician's decision to replan, as well as a weak correlation with a metric used to estimate the change in the outer body contour. However, none of these approaches use dosimetric information for decision making, and the comparative quality improvement of treatment using these two methods cannot be assessed. Further investigation, finding potential correlations between changes in in‐vivo images and PTV coverage and Organs at risk (OAR) dose for use in head‐and‐neck replanning is a topic of future study.

Automation is achieved using a scripting pipeline that downloads all patient EPID images collected on any given day on the Halcyon treatment machine. Even though this system currently downloads patient data at the end of the treatment day, this framework can be run at different time points if more urgent feedback is required. Currently, this tool analyzes the mean relative difference of pixels in a region of interest. Future work involves analyzing other image features, including using different regions of interest.

This approach also has some shortcomings. As the analysis is based on the first fraction, any changes from the time of simulation to the first fraction cannot be identified. In addition, any error in setup or treatment in the first fraction itself cannot be detected by this method, and hence any subsequent treatment fraction analysis would not be accurate. Also, any inconsistencies in the number of images, potentially due to beam interruption during the treatment, will have a falsely high GDSA mean, and hence were omitted during our analysis.

## 5 CONCLUSION

This novel and fully automated comprehensive QA tool can analyze every fraction of treatment to check for large errors as well as trend errors. This is possible due to automated download and analysis. The daily average and standard deviation give a measure of machine performance as well as patient variation in the given day. In addition, this tool can be ported over to other Varian Linacs to ensure comprehensive QA in other clinics as well, the only requirement being that the EPID arm is extended during the treatment.

## CONFLICT OF INTEREST

Dr. Casey Bojechko has received funding from Varian Medical Systems, grant number 20195180. The authors report no other conflicts of interest.

## AUTHOR CONTRIBUTIONS

Ananta Raj Chalise collected and analyzed the data and wrote the manuscript. Casey Bojechko designed the study, aided in data collection, oversaw data analysis and interpretation, and helped write the manuscript. All authors approved the final manuscript version.
